# Complex Autism Spectrum Disorder in a Patient with a Novel De Novo Heterozygous *MYT1L* Variant

**DOI:** 10.3390/genes14122122

**Published:** 2023-11-24

**Authors:** Silas Yip, Kristina Calli, Ying Qiao, Brett Trost, Stephen W. Scherer, M. E. Suzanne Lewis

**Affiliations:** 1 Department of Medical Genetics, University of British Columbia (UBC), Vancouver, BC V6H 3N1, Canada; silas.yip@bcchr.ca (S.Y.); kcalli@mail.ubc.ca (K.C.); yqiao@mail.ubc.ca (Y.Q.); 2 BC Children’s Hospital Research Institute, Vancouver, BC V6H 3N1, Canada; 3 Autism Spectrum Interdisciplinary Research (ASPIRE) Program, Vancouver, BC V6H 3N1, Canada; 4 The Centre for Applied Genomics and Program in Genetics and Genomic Biology, The Hospital for Sick Children, Toronto, ON M5G 0A4, Canada; brett.trost@sickkids.ca (B.T.); stephen.scherer@sickkids.ca (S.W.S.); 5 McLaughlin Centre and Department of Molecular Genetics, University of Toronto, Toronto, ON M5G 0A4, Canada

**Keywords:** *MYT1L*, autism spectrum disorder (ASD), neurodevelopmental disorder, single nucleotide variant (SNV), de novo, whole genome sequencing (WGS)

## Abstract

Autism spectrum disorder (ASD) comprises a group of complex neurodevelopmental features seen in many different forms due to variable causes. Highly impactful ASD-susceptibility genes are involved in pathways associated with brain development, chromatin remodeling, and transcription regulation. In this study, we investigate a proband with complex ASD. Whole genome sequencing revealed a novel de novo missense mutation of a highly conserved amino acid residue (NP_001289981.1:p.His516Gln; chr2:1917275; hg38) in the *MYT1L* neural transcription factor gene. In combination with in silico analysis on gene effect and pathogenicity, we described the proband’s phenotype and made comparisons with previously reported cases to explore the spectrum of clinical features in *MYT1L* single nucleotide variant (SNV) cases. The phenotype–genotype correlation showed a high degree of clinical similarity with previously reported cases of missense variants in *MYT1L,* indicating *MYT1L* as the causal gene for the observed phenotype in our proband. The variant was also predicted to be damaging according to multiple in silico pathogenicity predicting tools. This study expands the clinical description of SNVs on the *MYT1L* gene and provides insight into its contribution to ASD.

## 1. Introduction

ASD refers to a group of complex neurodevelopmental features characterized by persistent deficits in social communication and interaction, as well as restricted, repetitive behavioral patterns [1]. According to the Canadian Health Survey on Children and Youth (CHSCY), approximately 1 in 50 Canadians aged 1–17 years have been diagnosed with ASD as of 2019 [2]. ASD is a heterogenous disorder with varying genetic and environmental etiology. Studies have described a trend of impairment in cellular events such as progenitor cell proliferation, neuronal differentiation, dendrite outgrowth, and synaptogenesis [3]. Many ASD-susceptible genes are involved in chromatin remodeling and transcription regulation, which provides a potential basis for the dysregulated development observed for this disorder [3,4].

Transcription factors play an essential role in regulating the spatial and temporal gene expression patterns for brain development [3,5]. The Myelin Transcription Factor-1-like (*MYT1L*) gene encodes the MYT1L protein, a member of a zinc finger superfamily of neuronal transcription factors [6]. MYT1L has six characteristic zinc finger domains (zf-C2HC) containing three conserved cysteine and two conserved histidine residues within the consensus sequence C-P-X-P-G-C-X-G-X-G-H-X_7_-H-R-X_4_-C [7]. Studies have determined that *MYT1L* is primarily expressed in neuronal tissues in the fetal brain, with peaks during neurogenesis [8,9]. Across the central nervous system regions, *MYT1L* mRNA is upregulated when neurons begin to differentiate, predominantly during the post-specification phase of differentiation when cell populations are post-mitotic [10]. 

Several functions of *MYT1L* have been hypothesized in the literature. One study suggested that the expression of *MYT1L*, alongside transcription factors achaete-scute family basic helix-loop-helix transcription factor 1 (Ascl1) and POU class 3 homeobox 2 (Brn2), is critical to the efficient conversion of fibroblasts into mature functional neurons and induced neurons [11]. These three-factor induced neurons display functional properties such as generating action potentials and synapse formation [11]. This finding is further corroborated by knockdown studies of *MYT1L*, which has resulted in decreased synaptic transmission, axonal development, and neurite outgrowth in the cell [12]. Mall et al. found that knockdown of *MYT1L* in the mouse brain mimicked Notch gain-of-function phenotype and *MYT1L* depletion in primary post-mitotic neurons would de-repress non-neuronal programs and impair neuronal gene expression and function [13]. Shi et al. investigated the expression of *MYT1L* in rat oligodendrocyte precursors and found that *MYT1L* acts as an essential regulator of oligodendrocyte precursor cell (OPC) differentiation, where overexpression would promote differentiation and *MYT1L* knockdown inhibited OPC differentiation [14]. Their ChIP assays showed that MYT1L also bound to the Olig1 promoter and transcriptionally regulated Olig1 expression [14]. *MYT1L* loss-of-function (LoF) mutations often include frameshifts, deletions, and SNVs, which are predicted to decrease mRNA production [10]. De Rubeis et al. identified *MYT1L* as a gene that met a high statistical significance (0.05 < FDR ≤ 0.1), indicating that the gene has a 90% chance of being a true autism gene, while another study determined *MYT1L* to be an autism-associated gene with even higher confidence (FDR = 7 × 10^−8^) [15]. Human genetic studies have also strongly associated LoF variants of *MYT1L* with neurodevelopmental disorders such as autism spectrum disorder (ASD) [4,16,17]. Other common phenotypes associated with *MYT1L* LoF variants include intellectual disability (ID), obesity, developmental delay (DD), microcephaly, macrocephaly, and hypotonia [10,18,19]. This collection of clinical features describes *MYT1L* syndrome, otherwise known as 2p25.3 Deletion Syndrome or autosomal dominant intellectual disability-39 (OMIM #616521). Studies have emphasized the possibility of missense variants as LoF mutations and have implicated haploinsufficiency as the mechanism responsible for the observed *MYT1L* syndrome [9,18]. 

This report describes the phenotype of a proband with confirmed ASD who was genetically assessed through the Autism Spectrum Interdisciplinary Research (ASPIRE) Program at the British Columbia Children’s Hospital Research Institute located in Vancouver, Canada. Whole genome sequencing (WGS) revealed a novel de novo heterozygous variant in the *MYT1L* gene (NM_001303052.1:c.1548C > A; NP_001289981.1: p.His516Gln; chr2:1917275) [hg38]. We hypothesize this variant to be deleterious based on multiple in silico tools and characterize the observed phenotype of our proband using a comprehensive, standardized protocol. Furthermore, we describe the phenotype of the proband and draw comparisons with previously reported cases of similar de novo *MYT1L* SNVs to explore the variant’s phenotypic spectrum and to delineate its role in ASD. 

## 2. Materials and Methods

### 2.1. Recruitment and Data Collection

The proband initially presented for a Medical Genetics assessment of diagnosed ASD at the Provincial Medical Genetic Programme (PMGP) Clinic located at the British Columbia Women’s Hospital and Health Centre. The family was invited to participate in genomic testing through the ASPIRE program and ethics approved, informed consent to participate was obtained. The Medical Geneticist (M.E.S.L.) performed further clinical examination and genetic phenotyping. All relevant data and genetic information were systematically inputted into a confidential, secure online database known as ASDbase.

### 2.2. Whole Genome Sequencing and Variant Analysis

DNA was extracted from the peripheral blood of the proband and parents separately. Whole genome sequencing (WGS) was performed through ASPIRE’s iTARGET Autism Initiative project (http://www.itargetautism.ca/, accessed on 17 June 2022) in collaboration with The Center for Applied Genomics (TCAG; The Hospital for Sick Children, Toronto, ON, Canada) and the MSSNG project (https://www.mss.ng/, accessed on 17 June 2022). WGS was performed using the Illumina HiSeq X sequencing platform and reads were aligned to human reference genome hg38 (GRCh38). The internal pipeline for WGS and variant analysis has been described elsewhere [20,21]. The read depth of the WGS was >30X. Both VCF and BAM files were imported to the commercial software VarSeq (Golden Helix, Inc., Bozeman, MT, USA, https://www.goldenhelix.com, accessed on 22 June 2022) for SNV/insertion–deletion (indel) and copy number variant (CNV) analysis. A final list of CNVs was generated by the Binned Region Coverage (minimum 10 kb) and CNV algorithm in VarSeq (Golden Helix Inc., Bozeman, MT, USA). The SNVs/indels were filtered, annotated, classified, and interpreted by over 20 databases in VarSeq in combination with other databases such as OMIM, Decipher, ClinGen, ClinVar, HGMD, etc. The major parameters for quality control included Read Depth ≥ 10, Genotype Quality ≥ 20; minor allele frequency (MAF) ≤ 0.05 for homozygous recessive and compound heterozygous variants; MAF ≤ 0.01 for de novo, X-linked, imprinting gene, incidental findings (59 genes on the ACMG incidental finding list), and summarized gene lists from internal knowledge databases that include candidate genes involved in ASD and/or intellectual disability (ID), etc. The damaging missense prediction tools used in our analysis included Sorting Intolerant from Tolerant (SIFT) [22], Polyphen2HVAR [23], MutationTaster [24], Mutation Assessor [25,26], Functional Analysis through Hidden Markov Models (FATHMM) [27], and Rare Exome Variant Ensemble Learner (REVEL) [28]. A Combined Annotation Dependent Depletion (CADD) [29] score was also included in the evaluation. The SFARI gene score and EAGLE score [30], from the Simons Foundation Autism Research Initiative (SFARI) website (https://www.sfari.org, accessed on 22 June 2022) helped determine the relationship of *MYT1L* to ASD. The AlphaFold Structure Database version 1 November 2022 (https://www.alphafold.ebi.ac.uk, accessed on 28 March 2023) [31,32] was used to investigate the three-dimensional structure of the protein and its molecular interactions. 

### 2.3. Phenotypic Comparisons

A targeted search through the OVID Medline and GeneMatcher databases was conducted to identify similar published and unpublished *MYT1L* SNV cases. The OVID Medline search was conducted with the keyword: *MYT1L* and the subject headings: “Transcription Factors, Nerve Tissue Proteins, Intellectual Disability and *MYT1L*” were combined with the “AND” Boolean operator. All phenotypic categories included in our comparative analysis table (Supplementary Materials) were part of the clinical assessment performed at the PGMP and are based on the Human Phenotype Ontology (HPO) vocabulary. SNV cases were included if the authors provided a phenotypic description of the case patient and if the inheritance of the variant was determined to be de novo with confirmed parentage. Clinicians were contacted for more detailed phenotypic information in unpublished cases found in the GeneMatcher database. Phenotypic features that were not reported and had no indication of assessment or diagnosis were scored as “Not Reported” (NR) and were not included in the frequency calculation (Supplementary Materials). All data interpretation was based on the GRCh38/hg38 human genome assembly. The LiftOver tool on the UCSC Genome Browser (https://genome.ucsc.edu/index.html, accessed on 12 December 2022) was used to convert genomic coordinates from hg19 to hg38 for several published SNV cases in our analysis. Since genetic nomenclature was also inconsistent among different papers, Mutalyzer (https://mutalyzer.nl, accessed on 27 October 2023) was used to format all SNV mutations according to the Ensemble transcript NM_001303052 [33]. 

## 3. Results

### 3.1. Clinical History and Phenotype

The proband has one neurotypical brother and was born to a non-consanguineous 32-year-old mother and a 34-year-old father. A female first cousin was found to be a carrier of the *NF1* gene. There was no previous family history of ASD. Post-natal history was complicated, with feeding difficulty, poor suck, frequent vomiting, and “unusual sounds when feeding”. The proband experienced infantile muscular hypotonia. At age 3, the proband was diagnosed with ASD after meeting the DSM-IV criteria using standardized ADOS and ADI-R measures. Communication and social interaction scores on the ADOS exceeded the ASD cut-off. Intonation or verbalizations were not assessed since he only used single words during the assessment. Deficits in social development and atypical pattern behaviors were also observed, with scores meeting or exceeding the autism cut-off for the reciprocal social interactions, abnormalities in communication, and atypical behaviors categories on the ADI-R measure. He was also assessed with the Vineland Adaptive Behavior scale which reported mild to moderate adaptive delay and possible cognitive delay with overall functioning below a 2-year-old level, indicating severe global developmental delay (GDD). Although subsequent consultations with specialists at age 6 found intellectual impairment, formal testing for ID has yet to be performed. Additionally, the proband exhibited hyperactivity, poor eye contact, and stereotypic behavior. 

Clinical assessment was performed at the PMGP clinic at 4 years and 2 months. His height was 111.2 cm (95–97th percentile), his weight was 20.8 kg (95th percentile), and his occipital–frontal head circumference (OFC) was 53.5 cm (97th percentile), consistent with symmetric large stature. BMI was calculated to be 16.8 (83rd percentile) and not in the overweight or obesity category according to the CDC (https://www.cdc.gov/healthyweight/bmi/calculator.html, accessed on 21 August 2023). The calculator determines anyone aged 2–19 to be overweight if BMI is 85–95th percentile, while BMI greater than or equal to 95th percentile is considered obese. Macrocephaly was reported at birth. He exhibited occipital plagiocephaly, facial asymmetry, epicanthal folds, downward slanting palpebral fissures, a broad nasal root, depressed nasal bridge, thickening of the alae nasi, malar flattening, a high arched palate, and alveolar ridge overgrowth. The MSK exam revealed generalized hypotonia with ligamentous laxity, talipes equinovarus, significant calcaneovalgus, and metatarsus varus posturing. He has a history of toe walking which was resolved and was reported to have a tendency of “out-toeing”. He was observed to be non-verbal with hyper-nasal speech, drooling, and a hyperactive gag. He presented with abdominal colic at assessment and has a history of alternating diarrhea and constipation with hematochezia. His dysphagia was also characterized by frequent vomiting. During assessment of the nervous system, he presented with hyperacusis, pain insensitivity, and tactile defensiveness. He has also been formally diagnosed with epilepsy after three incidents of generalized tonic–clonic seizures with post-ictal lethargy. This was further evidenced by an abnormal EEG with a single fragment of generalized spike and wave discharges identified with the right frontal maximum. This result would be rare in individuals who have not previously had seizures and/or epilepsy. Starting the proband on lamotrigine has prevented further seizures. Self-injurious behavior, such as head banging, was also reported. At age 5, a head MRI revealed an abnormality in the white matter adjacent to the right occipital horn, suggesting incomplete myelination. All other system examinations were unremarkable. Routine genetic investigations such as karyotype, fragile X testing, and targeted fluorescence in situ hybridization (FISH) for 15q11-13, 22q11, 22q13, and 2q37.3 were normal. Blood biochemical analysis performed in 2005 found low blood ammonia, and subsequent testing conducted in 2006 and 2009 did not find abnormalities. Electrolytes, plasma amino acids, and urine organic acids were all unremarkable. 

### 3.2. Whole Genome Sequencing and Variant Analysis Findings

**Figure 1:**
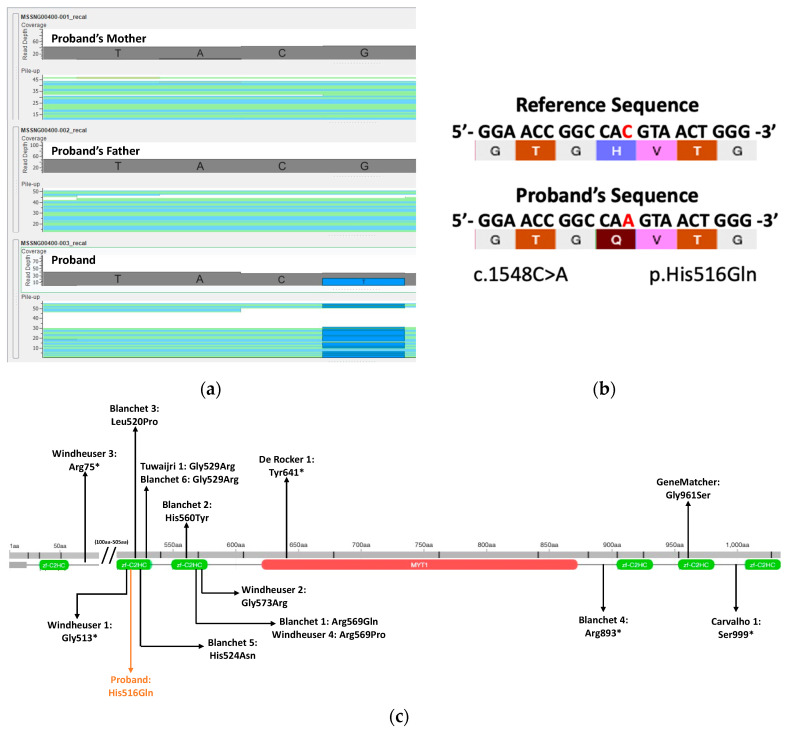
(**a**) BAM file comparison between proband and parental sequences determined a de novo *MYT1L* variant. (**b**) Schematic diagram of the *MYT1L* missense variant. (**c**) Location of proband and published variants on the *MYT1L* gene.

A novel heterozygous missense variant (NM_001303052.1:c.1548C>A; NP_001289981.1: p.His516Gln; chr2:1917275) [hg38] was identified on the *MYT1L* gene. As Figure 1a shows in the imported BAM files, the variant was determined to be de novo based on the comparison between proband and parental sequences and was further confirmed by Sanger sequencing in a clinical diagnostic laboratory. The allelic depth is 36 and the mismatch rate was determined to be 58.3%. As expected, based on its de novo status, this variant was not found in gnomAD. The variant was a substitution of a histidine residue for a glutamine residue on the second zf-C2HC domain (Figure 1b). Only one other de novo missense variant was identified in the GLTP gene; however, this was not an OMIM gene and had a PLI = 0.11. No other de novo missense or loss of function variants were identified in this proband.

In the VarSeq analysis, 5 out of 6 bioinformatic tools predicted the variant as damaging (Table 1). A deleterious effect was also predicted by the CADD PHRED score (24.3). SFARI also identified the *MYT1L* gene as a strong ASD candidate gene (Category 1, high confidence) and the EAGLE score (20.35) indicated *MYT1L* as a definitive ASD gene.

**Figure 2:**
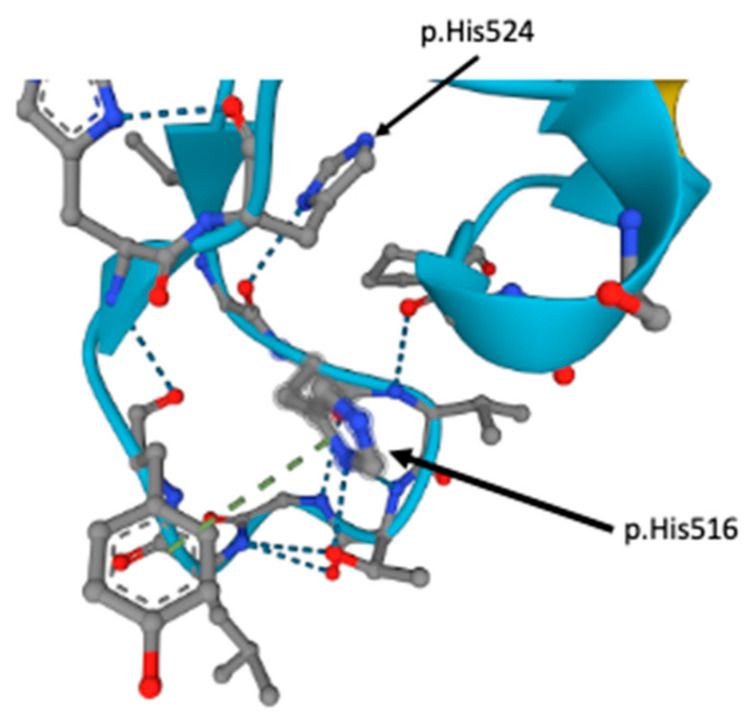
Three-dimensional structure of the zf-C2HC domain (UniProt Q9UL68) and two highly conserved histidine residues from the AlphaFold Protein Structure Database.

Based on genomic investigation, our variant (p.His516Gln) is located on a highly conserved residue on a functional folded protein loop that is part of the second zf-C2HC domain of the *MYT1L* gene. Given the structure of the histidine residues, it is predicted that the p.His516 residue participates in pi-stacking interactions through its aromatic rings (green dotted lines) and hydrogen bonding interactions (blue dotted lines) with adjacent residues (Figure 2). 

### 3.3. Phenotypic Comparisons of Patients with De Novo SNV MYT1L Variants

In total, fourteen de novo SNV *MYT1L* variants were included from the literature and the GeneMatcher databases (Supplementary Materials). Thirteen of these cases were found in published literature and one case was found from GeneMatcher. Table 2 shows the frequency of key characteristics among the cases. Two matched cases on GeneMatcher were not included in our comparisons as their phenotype descriptions were limited and did not provide enough information to make a comprehensive comparison. Although several attempts were made to request more phenotypic information from the clinicians of these cases, only one response was received from a collaborator in GeneMatcher. 

Speech delay was well documented and described in all individuals. Gross motor delay (sitting, walking, and/or standing) was reported in all individuals, with five individuals described to also have fine motor delay. Notably, ASD/autism was diagnosed in 36% of cases; 3 of the 5 individuals diagnosed with ASD/autism were found to be missense mutations on the second zf-C2HC domain similar to our proband [18,34], while two other variants were de novo nonsense mutations elsewhere on the gene [9,19]. ID/DD was reported in most cases (92%), with one individual from the GeneMatcher database not having intellectual involvement in their phenotype. Behavior abnormalities were present in 71.4% of cases but no generic behavioral trait was shared by all individuals. However, stereotyped movements were reported in both our proband and two other individuals, with one being a variant in the zf-C2HC domain [34]. Aggressive behavior, although not observed in our proband, was reported by three individuals with de novo mutations [19,35]. Self-injurious behavior was observed in our proband and two other individuals [19,34]. Facial dysmorphic features were common among cases (63.6%) but there were no shared distinctive dysmorphic features. Macrocephaly was observed in our proband and three other individuals, two of whom are missense variants in the zf-C2HC domains [19,34]. There is also a trend of larger stature and weight (with or without macrocephaly), with height and weight above the 90th percentile in three other missense variants and one LoF variant, similar to our proband, while one LoF case showed height at 86.5th percentile and weight >99th percentile (Supplementary Materials). Notably, our proband was not obese or overweight but of symmetric large stature. Further, he showed dysphagia and feeding problems. In comparison, the state of being overweight or obese was prevalent among the other cases with missense and LoF variants (67%), with five of these individuals also exhibiting hyperphagia. Two individuals had morphological changes detected with a brain MRI. One patient exhibited thinning of the corpus callosum while another exhibited cerebral atrophy [18]. Both of these variants were located within the two central zinc fingers (second and third zf-C2HC domains) of *MYT1L.*

## 4. Discussion

In this study, we describe the phenotype of an individual with an *MYT1L* missense variant using data collected through a comprehensive phenotype assessment protocol developed in the ASPIRE program. Using phenotypic data, WGS, and in silico analysis, this case report provides unique insights into the phenotypic spectrum caused by deleterious de novo *MYT1L* SNVs. Based on genomic analysis and the proband’s phenotypic similarity with existing cases, the *MYT1L* variant in our proband (p.His516Gln) is putatively deleterious and is the causal variant for the observed phenotypes. According to the SIFT, Polyphen2HVAR, MutationTaster, and Mutation Assessor tools, our novel *MYT1L* variant is deleterious (Table 1). Although FATHMM predicted a “tolerated” score, the high CADD PHRED score (24.3), SFARI gene score (Category 1, high confidence), and EAGLE score (20.35) further suggest the deleterious nature of our variant. CADD scores above 20 indicate that a variant is among the top 1% of all possible reference genome SNVs likely to have deleterious effects [29]. Our variant is currently not found in the gnomAD population database and the US (AOU) database. Our findings provide a valuable opportunity for further study into the molecular and cellular mechanisms that contribute to the pathophysiology of phenotypes associated with *MYT1L* variants. 

Based on the AlphaFold prediction, our variant resides on a highly conserved region of the gene and likely plays a role in forming a functional folded protein. Although studies have found that the second histidine residue (p.His524) is responsible for forming a functional zinc finger by coordinating metal ions [7], the first histidine residue (p.His516) is equally essential as it participates in pi-stacking interactions that stabilize a loop in the functional folded protein [36]. Subsequent studies have also discovered that both histidine residues are critical components for DNA recognition, given that mutation in either of the residues, including substitution with aromatic amino acids, result in the loss of the DNA binding function [37]. Mutations in these residues may disrupt transcription regulation on critical factors for neuron development and potentially provide an etiological explanation for neurodevelopmental disorders such as ASD and ID. Early studies have found that *MYT1L* binds to the target consensus motif, AAAGTTT, found on the *MYT1L* target promoter, β-retinoic acid response element (β-RARE) of the β-retinoic acid receptor (β-RAR) [6,38]. This is notable given that β-RAR is a critical factor that mediates neuron growth and differentiation [39]. While these findings provide more evidence suggesting that the *MYT1L* variant in our patient operates via loss of the function mechanism, the precise interactions between these transcription factors and target DNA sequence are still not fully understood [10,39], highlighting the need for further investigation in this area. Time-course analysis of chromatin accessibility and transcription binding during neurodevelopment may yield more information on this process [10]. 

LoF mutations in the *MYT1L* gene include microdeletions, frameshift, and SNVs [9,10]. Notably, missense mutations from prior clinical studies have been found to cluster in the highly conserved second and third central zinc finger domains [10]. Previous statistical analyses have found no significant differences in phenotype presentation between individuals with *MYT1L* microdeletion variants and those with *MYT1L* SNVs [9,18,19]. These previous findings support the hypothesis that SNVs, including missense mutations, can result in LoF and haploinsufficiency, which may disrupt critical gene expression during brain development. This is further corroborated by the significant phenotype overlap between our proband and both missense and LoF cases. Additionally, our variant’s clinical similarity with the missense variants in all features except obesity/overweight and hyperphagia suggests the *MYT1L* variant is the likely cause of the observed phenotype. 

Our proband was also not overweight/obese and did not exhibit hyperphagia, despite the clear association between *MYT1L* variants and obese/overweight features in the literature [18,35]. However, it is important to note that at least four other individuals were not obese/overweight in our comparisons, and although not obese, our proband did exhibit larger stature and weight for their age, similar to three other individuals with missense variants (Supplementary Materials). ASD was also found in a sizeable proportion of both LoF and missense cases. Among those with ASD, two were protein-truncating LoF variants [9,19], while AlphaFold predicted the other three missense mutations [18,34] to interfere with the DNA binding function. Most notably, all three cases were in the same domain as our variant. The missense variants from Tuwaijri and Alfadhel (p.Gly529Arg) and Blanchet et al. (p.Leu520Pro) both lie on a protein loop crucial for the folding of the second zf-C2HC domain [18,34], while the other missense variant from Blanchet et al. (p.His524Asn) is highly conserved and responsible for coordinating zinc ions within the second zf-C2HC domain. Any variation within the zf-C2HC domain may disrupt the protein structure, resulting in loss of function. In particular, the p.His524Asn variant from Blanchet et al. showed striking genomic and phenotypic resemblance with our proband. Blanchet et al.’s variant was located on the second highly conserved histidine residue that was required for the DNA binding function. Similar to our proband, this individual was assessed early and diagnosed with ASD/autism and presented with ID, DD, gross motor delay, speech delay, and hypotonia [18]. Unlike other described individuals, both cases exhibited no signs of hyperphagia and were not overweight/obese [18]. Similar to our proband, the possibility of incomplete myelination was also observed given that a brain MRI showed thinning of the corpus callosum, which was similar to our finding of incomplete myelination in the right occipital horn of the proband. A possible explanation may be due to decreased oligodendrocyte precursor differentiation, which may also be implicated as a cellular mechanism for ASD pathology [14]. However, functional testing would be required to confirm this. 

Our study has several important limitations to consider. Firstly, it lacks functional testing of the variant. We predicted the pathogenicity of the variant based on in silico bioinformatic tools, phenotypic comparisons, and structural analysis of the zinc finger domain. Secondly, there were varying degrees of comprehensiveness in phenotype reporting for *MYT1L* cases in the literature. As a result, certain phenotypes may have been missed and not included in our analysis. Thirdly, published cases in the literature did not indicate whether psychometric testing was used for the formal diagnosis of ASD. The comparisons were conducted with the assumption that those who were formally diagnosed met the DSM criteria for ASD and were not misdiagnosed. 

In conclusion, the findings from our genomic analysis and phenotypic comparisons support the hypothesis that our proband’s variant is deleterious and causes the observed phenotype of the proband. This case report expands the clinical description for *MYT1L* variants and emphasizes the role of *MYT1L* in the etiology of neurodevelopmental disorders such as ID and ASD. In addition, our report demonstrates the utility of WGS and the need for rigorous collection of phenotypic information when investigating ASD and other neurodevelopmental disorders. Although further functional testing of our variant is required, our analysis of the *MYT1L* variant highlights the importance of this gene in contributing to ASD.

## Figures and Tables

**Table 1:** Bioinformatic tools predict the *MYT1L* variant as damaging.

Tool	Converted Rankscore	Prediction
SIFT	0.912	Damaging
Polyphen2HVAR	0.97	Probably damaging
MutationTaster	0.588	Damaging
MutationAssessor	0.906	Predicted functional medium
FATHMM	0.605	Tolerated
REVEL	0.620	Likely causing disease

**Table 2:** Frequency of key phenotypic features in SNV cases.

Phenotypic Feature	Total Cases *	Missense Cases *	LoF Cases *	Proband
ASD/autism	5/14 (36%)	3/9 (33%)	2/5 (40%)	present
ID/DD	12/13 (92%)	8/9 (89%)	4/4 (100%)	present
Speech delay	14/14 (100%)	9/9 (100%)	5/5 (100%)	present
Motor delay	13/13 (100%)	9/9 (100%)	4/4 (100%)	present
Obesity/overweight	8/12 (67%)	4/7 (57%)	4/5 (80%)	not present
Hyperphagia/polyphagia	6/8 (75%)	4/5 (80%)	2/3 (67%)	not present
Macrocephaly	3/13 (23%)	2/8 (25%)	1/5 (20%)	present
Dysmorphic facial features	7/11 (64%)	3/6 (50%)	4/5 (80%)	present
Behavioral abnormalities	5/7 (71%)	2/4 (50%)	3/3 (100%)	present
Hypotonia	7/8 (88%)	5/6 (83%)	2/2 (100%)	present
Abnormal nervous system morphology	2/12 (17%)	2/9 (22%)	0/3 (0%)	present

* Numerator is the count of cases in which the feature is present. The denominator is the count of cases for which information on the presence or absence of that feature was available.

## Data Availability

Data presented in this study are available in the manuscript or are available upon written request from the corresponding author. The data are not publicly available due to confidentiality.

## Supplementary Materials

The following supporting information can be downloaded at: https://www.mdpi.com/article/10.3390/genes14122122/s1, Table S1: Phenotype comparisons between 14 similar MYT1L cases.
